# Supplementary material for: Full-color laser displays based on organic printed microlaser arrays
Source: Nat Commun. 2019 Feb 20;10:870. doi: 10.1038/s41467-019-08834-6 (PMC6382787; doi:10.1038/s41467-019-08834-6)
Supplement: Supplementary file 1 — Supplementary Information [file 41467_2019_8834_MOESM1_ESM.pdf]

Supplementary Information

Full-color laser displays based on organic printed microlaser arrays

Zhao et al.

Supplementary Figures

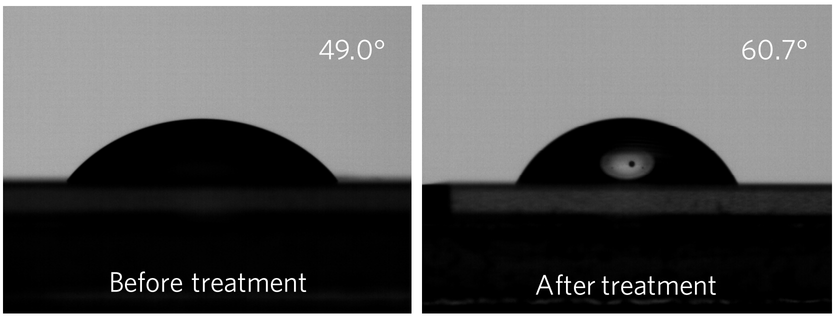

Supplementary Figure 1 Contact angle measurement of ink droplets on substrates before/after hydrophobic treatment. The contact angle of substrate was determined to be 49.0°, and increased to 60.7° through a hydrophobic treatment. This hydrophobic effect is the main drive to the formation of the spherical cap morphology of each droplet.

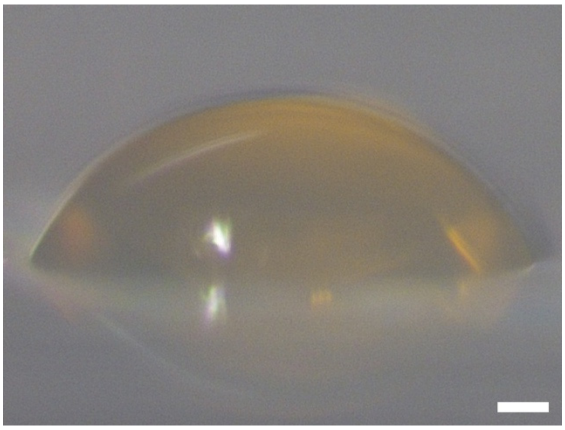

Supplementary Figure 2 Tilted optical microscopy image of an ink droplet upon dispersion. The scale bar is 40 μm. Benefiting from the hydrophobic effect, the ink droplet dispersed on substrates has a geometry of spherical cap.

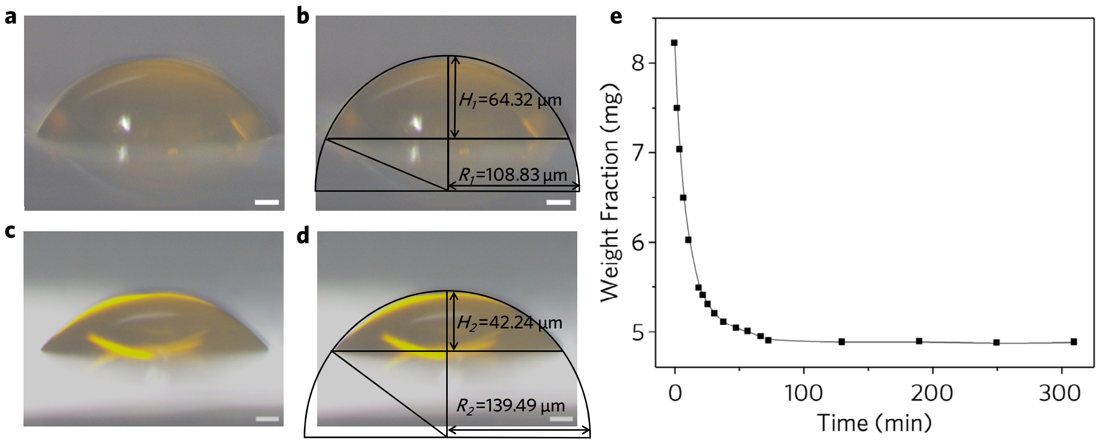

Supplementary Figure 3 Structural change of dispersed ink droplets upon drying. **a-d,** Volume shrinkage of a typical printed spherical cap. **a, b,** Tilted optical microscopy images of an ink droplet on a substrate without (**a**) / with (**b**) fitted geometric parameters. **c, d,** Tilted optical microscopy image of the dried droplet without (**c**) / with (**d**) fitted geometric parameters. All scale bars are 20 µm. **e,** Record masses of dispersed ink droplets at different time.

The dispersed droplets were dried in air for one day before optical characterization. In comparison with the ink droplets, the dried spherical caps exhibit obvious volume shrinkage. We compared the structural parameters of an identical spherical cap before and after drying (Supplementary Fig. 3). The ink droplet dispersed on the hydrophobic substrate exhibit a geometry of spherical cap (Supplementary Fig. 3a), and the height and radius can be determined to be 64.32 and 108.83 µm (Supplementary Fig. 3b), respectively. Assuming that the dispersed ink droplet has a perfect spherical cap geometry, we calculated the volume to be 1.135×10^6 µm^3 according to the formula $V = \pi H^2(R - H/3)$. Shown in Supplementary Fig. 3c-d are the

tilted optical microscopy images of the identical spherical cap after drying. There is an obvious reduction in height, while the base radius remains unchanged. Similarly, we obtained the geometric parameters and calculated a volume of 0.703 $\times 10^6$ μm^3. Thus, the volume shrinkage is (1.135-0.703)/1.135=38%.

In order to clarify the drying mechanism, we recorded the masses of dispersed ink droplets at different time, which are presented in Supplementary Fig. 3e. The mass decreases as time elapses in the first 1.5 hours and stabilizes in the subsequent several hours. The initial sharp reduction in mass should be attributed the water evaporation of inks because of negligible weight loss from other two constituents of the inks (BSA and glycerin). Therefore, the underlying drying mechanism of the ink droplets is ascribed to the evaporation of water contained in the inks.

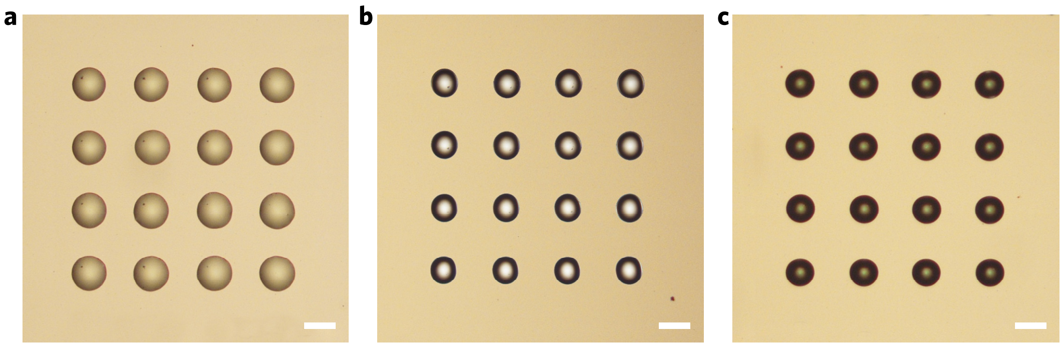

Supplementary Figure 4 Spherical cap shaped microlaser arrays prepared using various ink materials. **a-c**, Optical microscopy images of the microstructures printed with BSA aqueous solution (**a**), Araldite 506 epoxy resin (**b**), and NOA86 (**c**) on silver mirrors. All scale bars are 50 μm. These well-patterned spherical cap arrays indicate that the inkjet printing method is applicable for various ink materials.

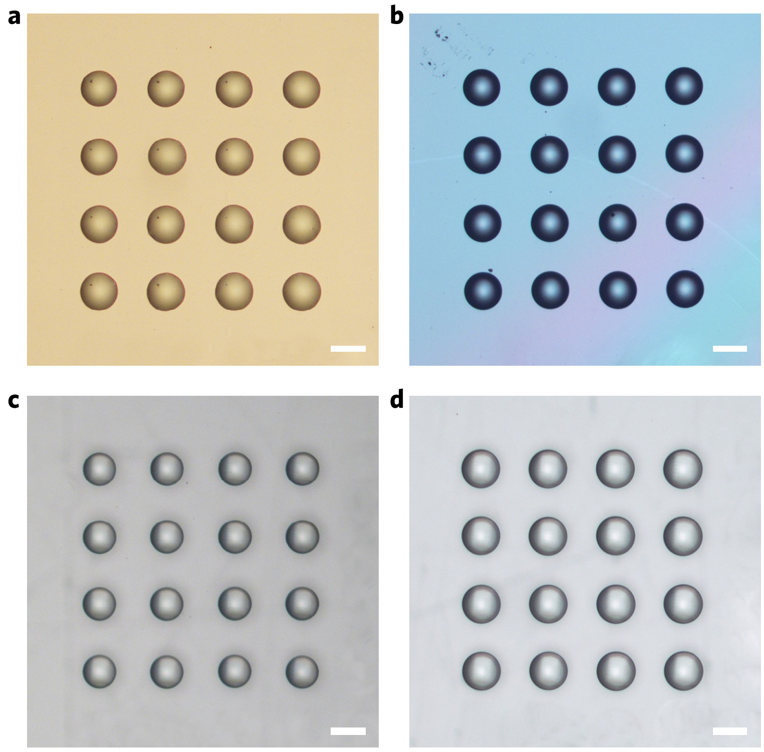

Supplementary Figure 5 Spherical cap shaped microlaser arrays prepared on different substrates. **a-d**, Optical microscopy images of spherical cap arrays printed with BSA aqueous solution on silver mirror (**a**), silicon wafer (**b**), glass (**c**), and PDMS (**d**). All scale bars are 50 μm. The results show that the inkjet printing can be applied on different substrates.

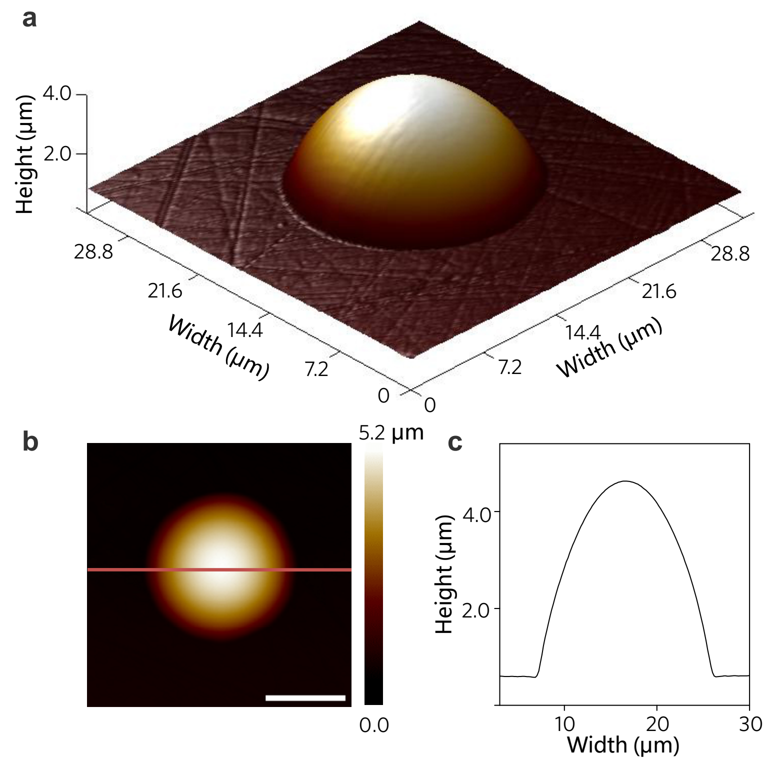

Supplementary Figure 6 Atomic force microscopy (AFM) images of a typical printed spherical cap. **a**, 3D-AFM image, **b**, 2D-AFM image and **c**, corresponding cross-sectional profile of the printed microstructure. The scale bar is 10 μm.

Supplementary Fig. 6 shows AFM images of a typical printed microstructure prepared using a vibration voltage of 1.0 V and a glass needle with a diameter of 10 μm. The as-fabricated microstructures possess a well-defined spherical cap geometry and perfectly smooth surface, which are favorable for WGM resonance.

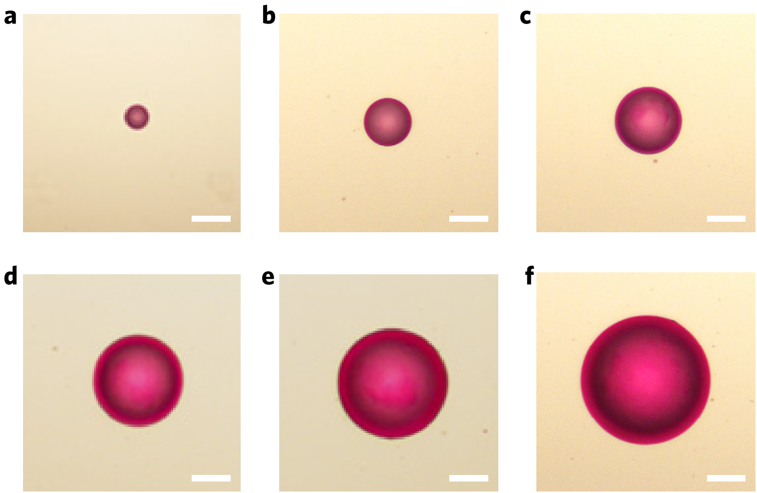

Supplementary Figure 7 Optical microscopy images of spherical cap shaped microlasers printed with glass needles of different tip diameters. **a**, 10 μm; **b**, 20 μm; **c**, 30 μm; **d**, 40 μm; **e**, 50 μm and **f**, 60 μm. All scale bars are 20 μm.

The base diameter of spherical cap strongly depends on the amount of ink solution released, which is controlled simultaneously by the diameter of the glass needle tip and the ultrasonic vibration strength. The size of the needle tip can be altered by direct replacement of the glass needle. As shown in Supplementary Fig. 7, the spherical caps were fabricated by printing RhB-doped BSA aqueous solution on a hydrophobic silver mirror substrate at a vibration voltage of 0.1 V. The base diameters of the resulting spherical caps were approximately 12.0, 22.6, 33.5, 45.3, 56.6 and 66.5 μm, when the applied tip diameter was 10, 20, 30, 40, 50 and 60 μm, respectively, revealing a monotonous increase with increasing needle tip diameter. Therefore, the size of the microcavities can be roughly controlled at the micrometer scale by changing the glass needle tip diameter.

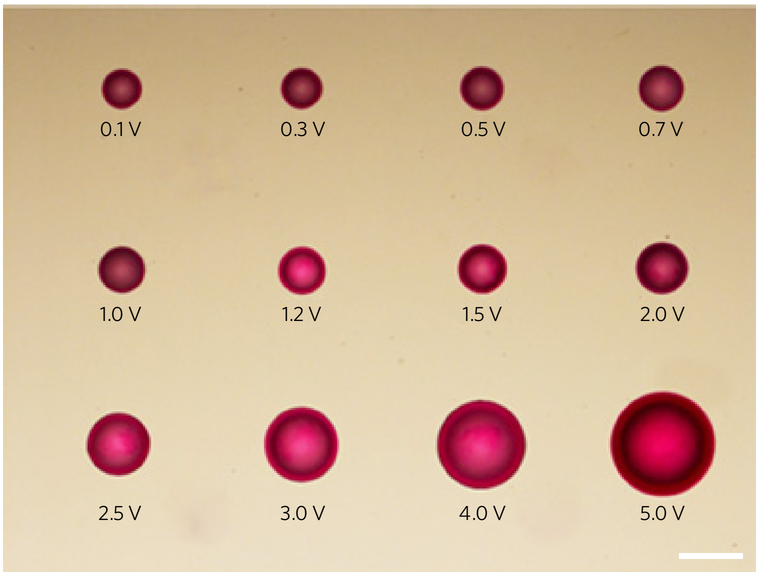

Supplementary Figure 8 Optical microscopy images of spherical cap shaped microlasers printed under different vibration strengths with an identical glass needle. The scale bar is 100 μm.

The spherical caps shown in Supplementary Fig. 8 were printed at different vibration voltages with an identical glass needle (tip diameter of 60 μm). The base diameters of the obtained spherical caps were 66.5, 67.4, 68.7, 69.5, 70.3, 71.8, 73.6, 80.1, 92.8, 110.2, 135.3, and 160.1 μm when the applied vibration voltages were 0.1, 0.3, 0.5, 0.7, 1.0, 1.2, 1.5, 2.0, 2.5, 3.0, 4.0, and 5.0 V, respectively, revealing a positive correlation between spherical cap size and the set vibration voltage. Therefore, the spherical cap size can be finely tuned at the hundred-nanometer scale by varying the vibration voltage.

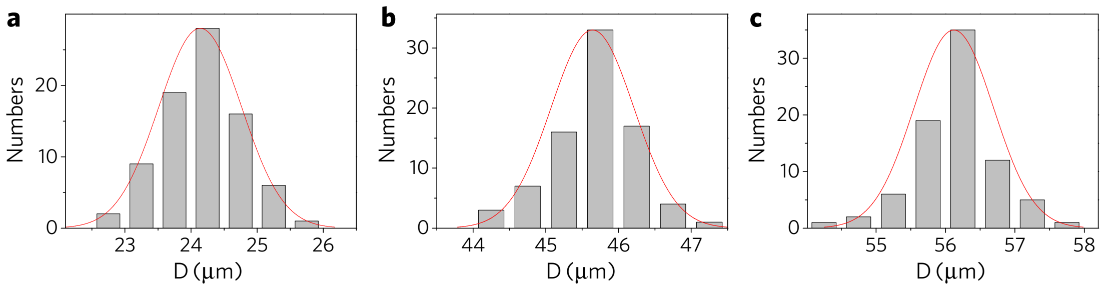

Supplementary Figure 9 Size distributions of spherical caps printed with glass needles of different tip diameters. **a**, 20 μm, **b**, 40 μm and **c**, 50 μm.

We fabricated three different 9 × 9 spherical cap arrays with glass needle tip sizes of 20 μm, 40 μm and 50 μm under the same vibration voltage (0.1 V). The base diameters of a total of 243 spherical caps were measured to investigate the size controllability and repeatability. Supplementary Fig. 9 presents the statistical histogram of the spherical caps. The mean diameter values were 24.1 (S.D. ±0.63), 45.6 (S.D. ±0.57) and 56.1 (S.D. ±0.57) μm for glass needle tip sizes of 20 μm, 40 μm and 50 μm, respectively, and the distribution can be fairly fitted by a Gaussian curve. These results all indicate that the printed spherical caps are highly reproducible, which is beneficial for constructing high-quality and large-area spherical cap microlaser arrays as display panels.

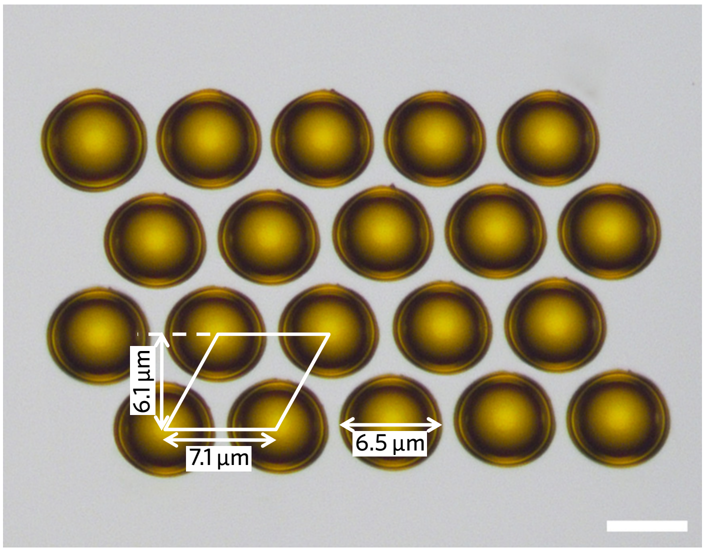

Supplementary Figure 10 Optical microscopy image of a closely packed microspherical cap pattern. The scale bar is 5 µm.

We prepared a closely-packed spherical cap array (Supplementary Fig. 10) using a glass needle with a tip diameter of 5 µm. In geometry, close-packing of equal spherical caps is a dense arrangement of congruent spherical caps in an infinite, regular arrangement (or lattice). Each spherical cap (with a base diameter of 6.5 µm) has six neighbors, and the center-to-center spacing of adjacent spherical caps is a simple honeycomb-like tessellation with a pitch (distance between spherical caps centers, 7.1 µm). Thus, the pack density should be the fraction of space occupied by the spherical cap in the lattice, which is exhibited in the Supplementary Fig. 10 as the white rhombus. The areas of individual spherical cap and rhombus are 33.2 μm^2 and 43.3 μm^2, respectively. So the highest packing density for spherical cap arrays printed with a 5 µm glass needle is 76.7%. We can fabricate a 140 × 164 spherical cap array on a

panel of 1 × 1 mm through the reported inkjet printing method, and the total number of the microcavities is 22960, demonstrating a high packing density.

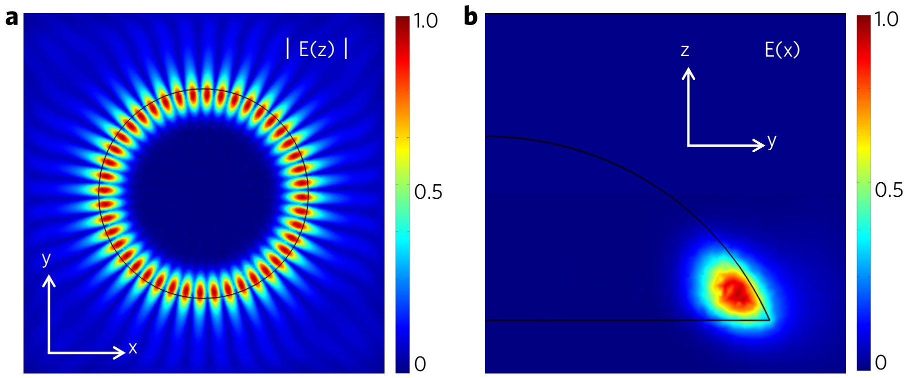

Supplementary Figure 11 Simulated 3D electric field distribution of resonant cavity modes in a typical spherical cap shaped microcavity. **a**, Electric field distribution inside a spherical cap in the horizontal plane. **b**, Electric field profile of the WGM in the vertical plane.

Supplementary Fig. 11 shows the electric field intensity distributions obtained using the finite-difference time-domain method. The energy of the photons is well-confined within the spherical cap, and the radial scattering into air is quite limited. The optical mode profiles along the edge of the spherical cap clearly show efficient light guiding, indicating a typical WGM-type resonance.

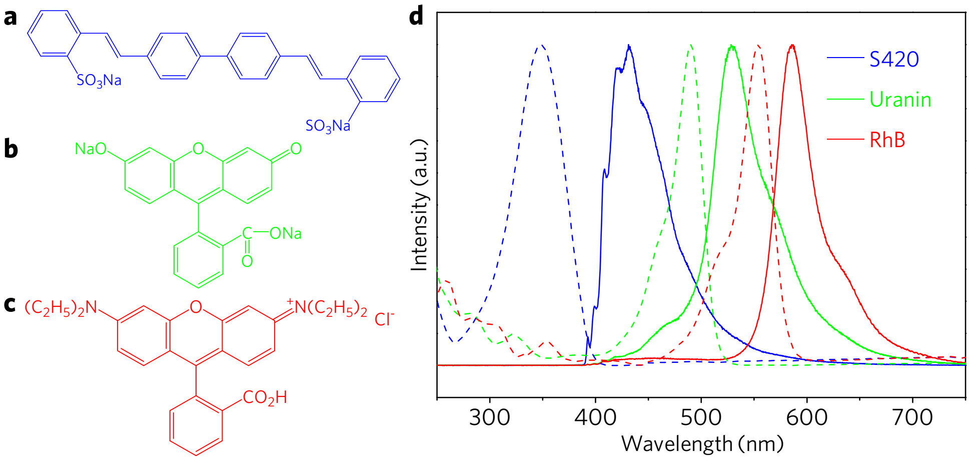

Supplementary Figure 12 Molecular structures and spectral data of the three laser dyes. **a-c**, Molecular structures of stilbene 420 (**a**), uranin (**b**), rhodamine B (**c**), respectively. **d**, Normalized absorption (dash) and PL spectra (solid) of the three laser dyes in aqueous solution. All concentrations of the three dyes are 0.01 mM.

Figure 12 presents the molecular structures and spectral data of the selected laser dyes. Because all three laser dyes can be excited with UV light (330-380 nm), we selected a 355-nm pulsed laser as the excitation source. The emission spectra span the full visible spectrum, offering an opportunity to achieve full-color laser output from a single pixel.

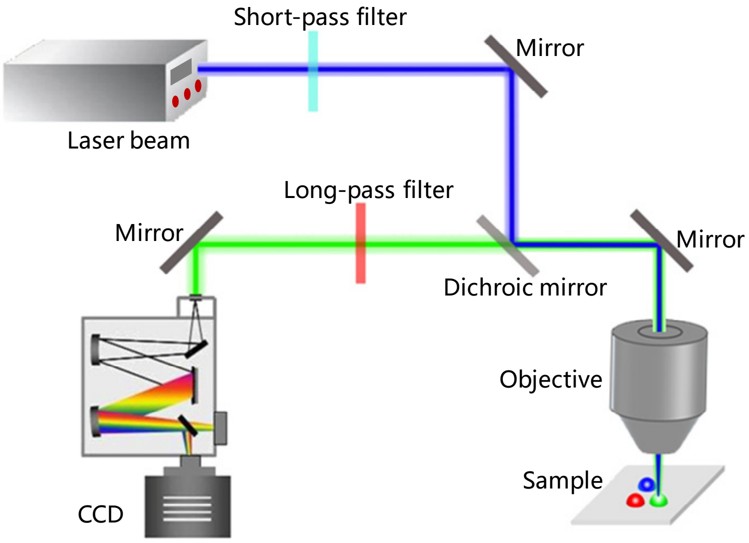

Supplementary Figure 13 Schematic illustration of the experimental setup for lasing characterization.

The lasing performances of the dye-doped spherical caps were examined using a custom microphotoluminescence system. A 355-nm femtosecond laser (Spectra-Physics, TOPAS) was employed as the excitation source, and the excitation energy was altered using neutral density filters. The PL signal was collected with an objective (Nikon CFLU Plan, 20×, N.A.=0.5). After passing through the corresponding filters (400-nm long-pass), the collected emissions were dispersed with a grating (1200 G mm^{-1}) and the recorded using a thermal-electrically cooled CCD (Princeton Instruments, ProEm 1600B).

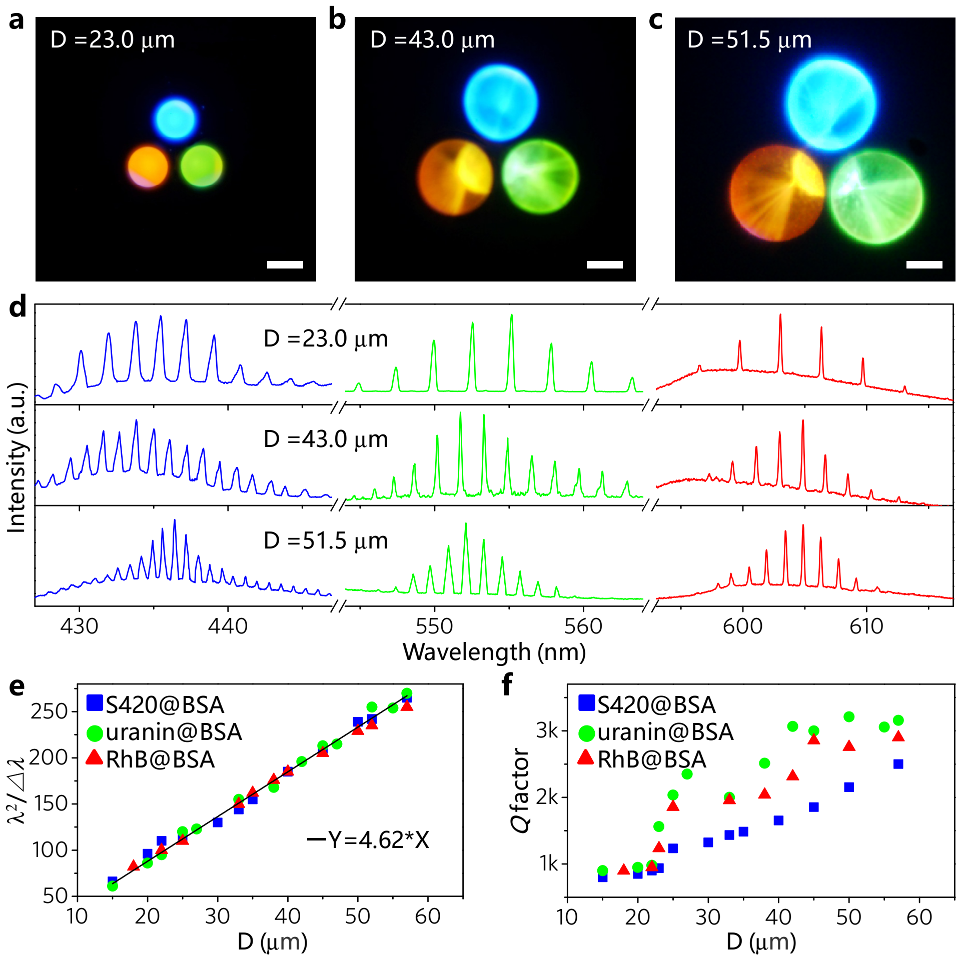

Supplementary Figure 14 Lasing characteristics of individual RGB pixels of different sizes. **a-c**, PL images of printed RGB spherical caps with different diameters. The RGB pixels were pumped by a pulsed laser beam. The beam waist was adjusted to be sufficiently large to simultaneously excite all three spherical caps. All scale bars are 20 μm. **d**, PL spectra of the RGB pixels with different diameters. **e**, Relationship between $\lambda^2/\Delta\lambda$ and the diameter of the spherical caps D. The black line is a fit to the function $\lambda^2/\Delta\lambda = n\pi D$, which verifies the WGM cavity property of the printed spherical caps. **f**, Plot of the experimental quality factor *vs* spherical cap base diameter. All quality factors are higher than 10^3, indicating that these RGB pixels can generate excellent laser emission to satisfy the requirement of laser displays.

By altering the size of the spherical caps, we can tune the lasing characteristics of these flexible RGB pixels, including the mode spacing and the quality (Q) factor. Supplementary Fig. 14a-d presents PL images and lasing spectra of three distinct RGB pixels with different sizes. Bright emission was observed along their ring-shaped boundaries, which is the typical characteristic of the formation of WGM resonance. All spectra show a series of peaks with different values of mode spacing ($\Delta\lambda$). According to the WGM theory, the mode spacing $\Delta\lambda$ and the base diameter D should satisfy the equation $\lambda^2/\Delta\lambda = n\pi D$, where λ is the wavelength of the guided light, and n is the group refractive index, respectively. On the basis of the experimentally measured relationship between $\lambda^2/\Delta\lambda$ and D (Supplementary Fig. 14e), $n = 1.47$ was obtained, consistent with the intrinsic refractive index of the BSA polymer (1.47). This result indicates that the optical WGM modes are tightly confined in the polymer matrix. The measured quality factors of the cavities were on the order of 10^3, which is quite high for organic resonators (Supplementary Fig. 14f)[1]. The mode numbers decrease with increasing of spherical cap base diameter, indicating that a single-mode laser, which is considered the optimal source for laser displays, is obtainable by further shortening of the cavity length.

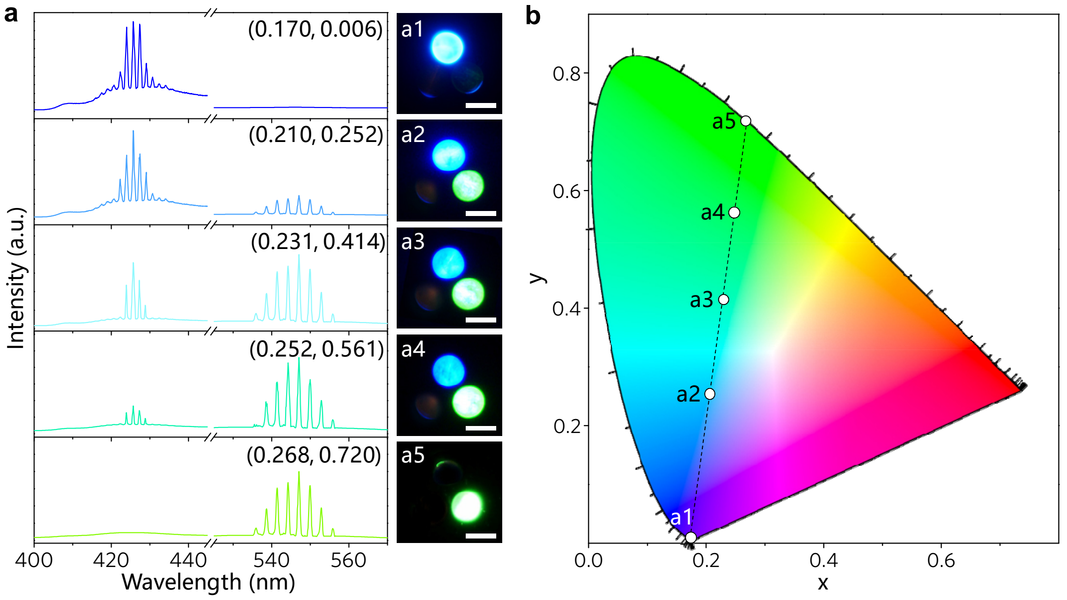

Supplementary Figure 15 Dual-color tunable lasing from a typical pixel. **a**, Lasing emission spectra and corresponding PL images of a single pixel under laser excitation at different positions. The numbers show the CIE1931 coordinates calculated from the corresponding spectra. All scale bars are 10 μm. **b**, Chromaticity of the lasing peaks extracted from the spectra in **a**, shown as five white circles.

As shown in Supplementary Fig. 15a, the relative intensities of the blue laser and green laser can be adjusted by modulating the position and power of the pumping beam, indicating the possibility of generating lasing emission consisting of blue and green light components in varied proportions. The reported CIE1931 coordinates were calculated from the corresponding spectra. Figure 15b shows the obtained five coordinates on a CIE1931 color diagram. All five colors are located along the dashed line, which is defined by connecting the spots extracted from the blue (Fig. 15a, top) and green (Fig. 15a bottom) lasing spectra. Similarly, other light combinations such as

red-blue and red-green light are available. Thus, more colors can be obtained by precisely mixing the three primary colors in appropriate proportions by adjusting the manner of excitation.

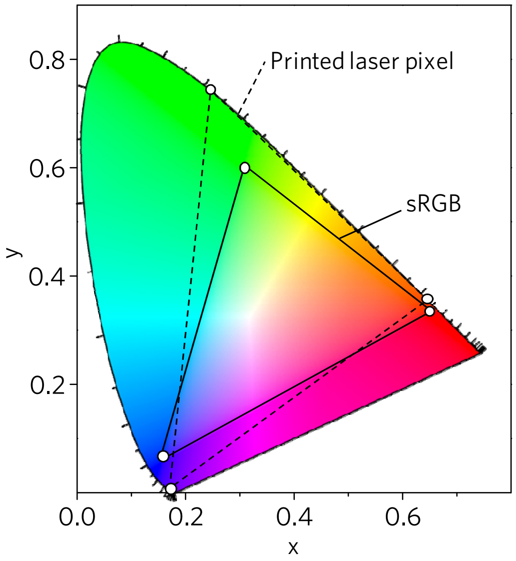

Supplementary Figure 16 Comparison of the color gamut between an individual printed RGB pixel (dashed) and the standard RGB (sRGB) color space (solid).

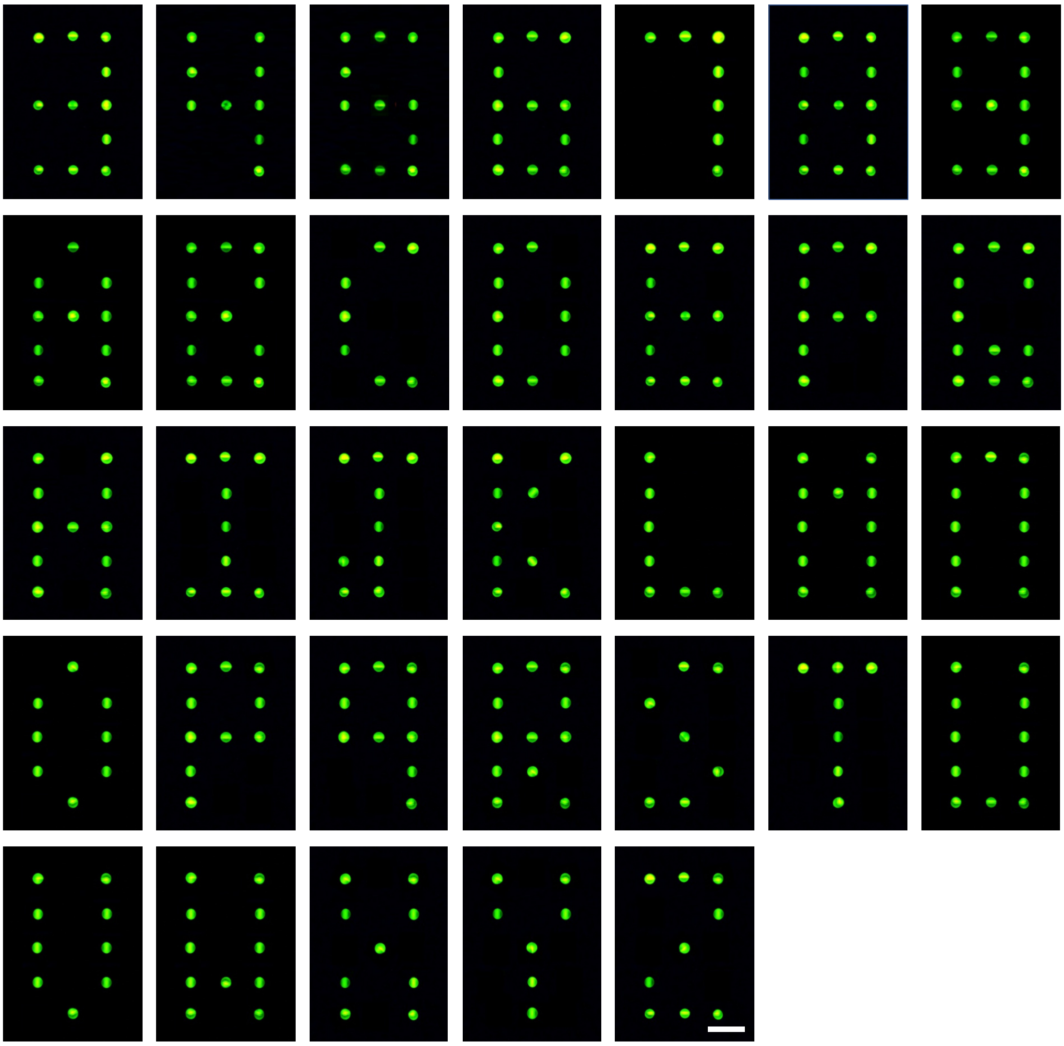

Supplementary Figure 17 PL images of a 3 × 5 pixel array for the display of different characters. The scale bar is 200 μm.

Supplementary Fig. 17 presents green laser displays of different characters created by selectively exciting specified uranin-doped spherical caps on a 3 × 5 pixel array identical to the one shown in Fig. 4b. This figure demonstrates the ability to display arbitrary numbers and characters using the as-developed display system, which can be applied to show desired text information.

Supplementary Tables

Supplementary Table 1 Structural parameters of spherical cap shaped microlasers prepared under different conditions.

spherical cap base diameter (μm)		needle tip size (μm)					
		10	20	30	40	50	60
vibration voltage (V)	0.1	12.0	22.6	33.5	45.3	56.6	66.5
	0.3	12.8	23.2	34.2	45.8	57.4	67.5
	0.5	14.3	23.9	35.1	46.6	58.2	68.7
	0.7	16.6	25.6	36.8	47.1	60.0	69.5
	1.0	18.2	27.5	38.7	49.5	62.6	70.3
	1.2	19.3	29.6	39.4	50.3	64.2	71.8
	1.5	20.6	32.4	41.1	52.5	67.2	73.6
	2.0	23.5	35.4	45.9	54.6	73.5	80.1
	2.5	25.6	39.7	49.3	60.2	80.5	92.8
	3.0	29.4	45.2	55.4	70.2	95.8	110.2
	4.0	35.5	58.6	75.3	96.2	120.6	135.3
	5.0	40.0	70.5	95.4	121.5	145.4	160.1

Experimentally, we obtained well-defined spherical caps ranging from ~12.0 to 160.1 μm in diameter by printing a single droplet on the hydrophobic substrate. The outstanding size controllability ensures good mass production of spherical cap shaped microlaser arrays for display panels.

Supplementary Table 2 CIE1931 chromaticity coordinates of the RGB pixel and standard RGB.

Sample		Chromaticity coordinates in CIE1931	
		x	y
RGB pixel	R	0.649	0.345
	G	0.268	0.720
	B	0.170	0.006
sRGB	R	0.640	0.330
	G	0.300	0.600
	B	0.150	0.060

Supplementary Table 3 CIE1976 chromaticity coordinates of the RGB pixel and standard RGB.

Sample		Chromaticity coordinates in CIE1976	
		u'	v'
RGB pixel	R	0.444	0.531
	G	0.096	0.584
	B	0.249	0.020
sRGB	R	0.451	0.523
	G	0.125	0.563
	B	0.175	0.158

Supplementary Notes

Supplementary Note 1

The saturation and contrast of laser displays are inherently higher than those of current display technologies because colors generated by combining RGB laser sources with narrow spectral lines are more vivid than those generated by broadband light sources[2]. Supplementary Fig. 16 illustrates the CIE1931 chromaticity color diagram of a printed RGB laser pixel and the standard RGB (sRGB), which is widely used in industry[3]. The corresponding chromaticity coordinates (x, y) are given in Supplementary Table 2.

To compare the color gamut of our RGB pixel with that of the sRGB, we calculated their color gamuts after converting to a perceptually uniform color space (CIE1976). The chromaticity coordinates (u', v') in CIE1976 summarized in Supplementary Table 3 were obtained from Supplementary Equation 1[4].

$$u' = \frac{4x}{-2x + 12y + 3}, \quad v' = \frac{9y}{-2x + 12y + 3} \tag{1}$$

The three sets of color coordinates constitute a triangle in the CIE color diagram. All colors in the triangle can be displayed by proper mixing of the three primary colors. The area of the RGB triangle (A) can be calculated according to Supplementary Equation 2[5].

$$A = \frac{(u'_r - u'_b)(v'_g - v'_b) - (u'_g - u'_b)(v'_r - v'_b)}{2} \tag{2}$$

The areas of the RGB pixel and sRGB were calculated to be 0.0941 and 0.0649, respectively. Therefore, the printed RGB laser pixel covers 45% more perceptible colors than the standard RGB[6].

Supplementary References

1. Wei, C. *et al.* Controlled self-assembly of organic composite microdisks for efficient output coupling of whispering-gallery-mode lasers. *J. Am. Chem. Soc.* **137,** 62-65 (2015).
2. Fan, F., Turkdogan, S., Liu, Z., Shelhammer, D. & Ning, C. Z. A monolithic white laser. *Nat. Nanotechnol.* **10,** 796-803 (2015).
3. Süsstrunk, S., Buckley, R. & Swen, S. Standard RGB color spaces. *Proc. IS&T/SID 7th Color Imaging Conference* 127-134 (1999).
4. Luo, M. R. The quality of light sources. *Color. Technol.* **127,** 75-87 (2011).
5. Luo, Z., Chen, Y. & Wu, S. T. Wide color gamut LCD with a quantum dot backlight. *Opt. Express* **21,** 26269-26284 (2013).
6. Hunter, R. S. Photoelectric color difference meter. *J. Opt. Soc. Am.* **48,** 985-995 (1958).
