# Supplementary material for: Full-color laser displays based on organic printed microlaser arrays
Source: Nat Commun. 2019 Feb 20;10:870. doi: 10.1038/s41467-019-08834-6 (PMC6382787; doi:10.1038/s41467-019-08834-6)
Supplement: Supplementary file 3 — Description of Additional Supplementary Files [file 41467_2019_8834_MOESM3_ESM.docx]

**Description of Additional Supplementary Files**

File Name: Supplementary Movie 1

Description: **An animation revealing the dynamic display of RGB numbers 0-9 with a fast refresh rate.** The movie presents an animation recording the dynamic display with a fast refresh rate. Blue, green and red numbers 0-9 were successively displayed on an identical panel composed of a pixelated 3 × 5 microlaser array. Each frame image in the animation was obtained by consecutively scanning the excitation beam along a corresponding path to light up pixels at specific locations. Frame-by-frame fast scanning enabled rapid switching between the displays of different numbers. The fluorescence lifetime of organic dyes (on the order of nanoseconds) is much shorter than the persistence of human vision, thus enabling rapid refreshing of the pixels on the panel to achieve fast dynamic display. The results verify that accurately positioned RGB microlaser arrays can be favorably applied in the display of movies with excellent color expression.
